# Nanoparticles Loaded with *Lippia graveolens* Essential Oil as a Topical Delivery System: In Vitro Antiherpetic Activity and Biophysical Parameters Evaluation

**DOI:** 10.3390/pharmaceutics17101286

**Published:** 2025-10-02

**Authors:** Nancy Nallely Espinosa-Carranza, Rocío Álvarez-Román, David A. Silva-Mares, Luis A. Pérez-López, Catalina Leos-Rivas, Catalina Rivas-Morales, Juan Gabriel Báez-González, Sergio Arturo Galindo-Rodríguez

**Affiliations:** 1 Laboratorio de Nanotecnología, Facultad de Ciencias Biológicas, Universidad Autónoma de Nuevo León, San Nicolás de los Garza 66455, Nuevo León, Mexico; nancy.espinosacrr@uanl.edu.mx (N.N.E.-C.); catalina.leosrs@uanl.edu.mx (C.L.-R.); catalina.rivasmr@uanl.edu.mx (C.R.-M.); 2 Departamento de Química Analítica, Facultad de Medicina, Universidad Autónoma de Nuevo León, Monterrey 64460, Nuevo León, Mexico; rocio.alvarezrm@uanl.edu.mx (R.Á.-R.); david.silvamr@uanl.edu.mx (D.A.S.-M.); luis.perezlp@uanl.edu.mx (L.A.P.-L.); 3 Departamento de Alimentos, Facultad de Ciencias Biológicas, Universidad Autónoma de Nuevo León, San Nicolás de los Garza 66455, Nuevo León, Mexico; juan.baezgn@uanl.edu.mx

**Keywords:** nanoparticles, essential oils, HVS-1, antiviral, *Lippia graveolens*

## Abstract

**Background/Objectives**: The skin is a protective barrier against pathogens such as herpes simplex virus type 1 (HSV-1), which causes recurrent and highly prevalent skin infections worldwide. The increasing resistance of HSV-1 to conventional treatments has driven the search for new therapeutic alternatives. In this context, the essential oil of *Lippia graveolens* (EOL) has demonstrated promising antiviral activity; however, its high volatility limits direct skin application. To overcome this, polymeric nanoparticles (NPs) loaded with EOL were developed to improve its availability and antiviral efficacy. **Methods**: Nanoformulations were prepared by nanoprecipitation, and their antiviral activity against HSV-1 was evaluated using the plaque reduction assay. The effect of the nanoformulations on skin barrier integrity was assessed using an ex vivo porcine skin model and non-invasive techniques. **Results**: The NP-EOL exhibited physicochemical properties favorable for skin deposition, including a particle size around 200 nm, a polydispersity index of ≤ 0.2, and negative zeta potential. Moreover, NP-EOL showed 1.85-fold higher antiviral activity against HSV-1 compared with free EOL, while also reducing cytotoxicity in Vero cells. **Conclusions**: Results demonstrated that the NPs promoted skin hydration without altering pH or transepidermal water loss, suggesting they do not disrupt skin homeostasis. This study supports the potential of NP-based systems as effective topical delivery vehicles for EOL, representing a promising therapeutic alternative against HSV-1 skin infections.

## 1. Introduction

The skin plays a crucial role in protecting the body against environmental factors. This protective function is primarily attributed to the stratum corneum (SC), the outermost layer of the epidermis. The SC is composed of corneocytes that are surrounded by ceramides, fatty acids, and cholesterol; together they form a lamellar hydrophobic structure that limits transepidermal water loss and prevents the entrance of harmful substances and pathogenic microbes. In addition, various components such as enzymes, protease inhibitors, immune cells (e.g., Langerhans cells), and antimicrobial peptides contribute to the homeostasis of the SC barrier [1]. However, the integrity of the skin barrier can be compromised by diverse factors such as trauma, burns, skin disorders, and underlying medical conditions leading to cutaneous infections caused by pathogens (i.e., bacteria, fungi, and viruses) that penetrate this barrier [2].

Herpes simplex virus type 1 (HSV-1) is an enveloped, double-stranded DNA virus that causes recurrent vesicular eruptions, most commonly in the orofacial region (i.e., cold sores) [3]. HSV-1 is highly contagious, and it is estimated that 3.7 billion people under the age of 50 are infected with the virus. The main antiherpetic treatments used to accelerate the healing of blisters and relieve symptoms such as pain, itching, or burning sensations are acyclic nucleosides, including acyclovir and derivatives (i.e., famciclovir and valacyclovir) [4]. Depending on the severity of the symptoms and the host’s immune status, the antiviral therapy can be administered either systemically or topically [5,6]. However, one of the central challenges associated with oral antiviral treatments is their low bioavailability caused by the limited absorption of the drug through the gastrointestinal tract. In the case of acyclovir, some patients have exhibited absorption rates as low as 10–27% of the administered dose. This low bioavailability requires higher doses, which can lead to adverse effects such as nausea, vomiting, headache, irritability, and even renal injury [7]. On the other hand, for topical treatment, acyclovir is not a good candidate for passive permeation due to its polarity (logP = −1.76 at pH 7), which limits its partitioning into the lipid matrix of the SC [8]. In addition, resistance of HSV-1 to these antiviral treatments has become a significant concern, particularly in immunocompromised patients undergoing long-term therapies [9]. Currently, there is growing interest in the development of new antiviral agents with alternative mechanisms of action that can replace or complement existing therapies, for example, essential oils (EOs).

EOs are complex mixtures of terpenoids, ketones, aldehydes, and esters that have been approved for use in the food, cosmetic, and pharmaceutical industries [10]. Various studies have demonstrated their beneficial biological properties, including inhibitory activity against HSV-1 [11,12]. Specifically, oregano has shown promising antiviral properties. The chemical composition of oregano EO includes hydrocarbon sesquiterpenes (β-caryophyllene), hydrocarbon monoterpenes (ρ-cymene, β-thujene), and oxygenated monoterpenes (carvacrol and thymol), the latter being the major constituents as they represent about 20–60% of the total composition [13,14,15]. The antiviral activity of oregano EO (*Lippia graveolens*) against HSV-1 was reported by Pilau et al. [16], who determined a 50% inhibitory concentration (IC_50_) of 99.6 μg/mL. Later, another study demonstrated that specific components of oregano EO like β-caryophyllene (IC_50_ = 0.25 μg/mL), ρ-cymene (IC_50_ > 0.1%, equivalent to >1000 μg/mL), thymol, and carvacrol (IC_50_ = 7 μM, equivalent to 1.1 μg/mL) display antiviral activity against the virus [17]. Although EOs hold great potential as therapeutic alternatives, their use is limited due to their physicochemical characteristics. For example, they are volatile at room temperature and susceptible to degradation in the presence of oxygen, light, and humidity, moreover their application in aqueous-based formulations is challenging because of their nonpolar nature [18].

To approach these limitations, nanoencapsulation can result in an efficient strategy to deliver active compounds including EO whose chemical characteristics influence their usage. Specifically, polymeric nanoparticles (NPs) have been extensively studied for topical use because their nanometric size increases residence time and enhances direct contact with the skin. Moreover, their polymeric composition enables the controlled release of EO onto the stratum corneum (SC), improving their availability and consequently, the efficacy of the active compounds [19]. Also, it has been shown that NPs with sizes around 200 nm tend to accumulate in hair follicles and skin folds [20]. For these reasons, NPs represent a promising tool for the topical delivery of EOs in the treatment of HSV-1 related infections. In a study by Kalita et al. [21], chloramphenicol and lemongrass EOs were co-encapsulated in a poly-ε-caprolactone NPs for topical application in the treatment of burns co-infected with bacteria and fungi. The nanoformulation exhibited enhanced in vitro antimicrobial activity against 22 microbial pathogens. Additionally, the NPs facilitated penetration into the injured dermal tissue and accelerated the wound healing process.

Therefore, the aim of this study was to evaluate the in vitro antiviral activity against HSV-1 using a polymeric NP formulation loaded with *Lippia graveolens* EO (EOL). The investigation also measured the effect of the NPs on the biophysical properties of the skin, in order to determine the potential use of the formulation as a topical treatment for dermal infections with HSV-1.

## 2. Materials and Methods

### 2.1. Materials

*Lippia graveolens* was collected in Coahuila, Mexico. The chemical composition and the proportion of each component of the EO were analyzed by González-Moreno et al., [22] using a gas chromatography–mass spectrometry (GC-MS) and gas chromatography with flame ionization detection (GC-FID). The Vero cell line (ATCC CCL-81) and the HSV-1 strain KOS (ATCC VR-1493D) virus were kindly donated from the Department of Genetics, School of Medicine, UANL. Dimethylsulfoxide (DMSO) and carvacrol (CRV) (≥98% grade GC) were obtained from Sigma Aldrich (St. Louis, MO, USA). Methanol was purchased from Fisher Chemical (Waltham, MA, USA), while acetone and isopropyl alcohol were obtained from Tedia (Fairfield, OH, USA). All solvents were of analytical grade. Distilled water was obtained from a Milli-Q water purification system (Boston, MA, USA). The nanoparticle-forming polymer, Eudragit^®^ L100 (copolymer of methacrylic acid-methyl methacrylate (1:1)), was kindly obtained from Helm (Naucalpan, State of Mexico, Mexico). Dulbecco Modified Eagle Medium (DMEM), fetal bovine serum (FBS), and the rest of the reagents used for the in vitro tests were from Gibco, Grand Island, NY, USA.

### 2.2. Preparation and Characterization of EO-Loaded NP

The NPs were prepared by the nanoprecipitation method as described by Fessi et al. [23]. Briefly, an organic phase was prepared with 25 mg of Eudragit^®^ L100 in 15 mL of acetone–isopropanol mixture (1:1). Then, 50 mg of EOL was added to the solution. Subsequently, the organic phase was injected using a syringe into the aqueous phase containing 20 mL of distilled water under magnetic stirring. Diffusion of the organic phase into the aqueous phase induced the aggregation polymer and encapsulation of EOL into nanoparticles (NP-EOL). Finally, the organic solvents were removed through two purification techniques: (i) dialysis using regenerated cellulose membrane Spectrum/Por®4 (Spectrum Laboratories, Rancho Dominguez, CA, USA) and (ii) evaporation under reduced pressure (Laborota 4003 control, Heidolph Instruments, Schwabach, Germany). EOL-loaded NPs purified by dialysis were identified as NP-EOL-D, while those purified by evaporation were identified as NP-EOL-R. Unloaded NPs, without EOL, were obtained following the same procedure described above. Similarly, the NPs without EOL purified by dialysis were identified as NP-BCO-D, and those purified by evaporation as NP-BCO-R.

The physicochemical characterization of formulations was carried out in terms of particle size, polydispersity index (PDI), zeta potential, and pH (Conductronic, pH120 model, Puebla, Puebla, Mexico). The mean particle size and PDI were measured at a 90-degree scattering angle using dynamic light scattering, while the zeta potential was measured by laser Doppler microelectrophoresis. Both measurements were performed using a Zetasizer Nano-ZS90 (Malvern Instruments, Worcestershire, UK), in triplicate at 25 °C. In addition, morphological examination of NPs was performed using a scanning electron microscope (JEOL JSM-7401F, Kyoto, Japan). Samples of dried NPs were dispersed in water and then, drops of the dispersion were placed on metallic studs and coated with gold.

The NP-EOL-R and the NP-EOL-D formulations were stored at 25 ± 2 °C under 40–60% relative humidity conditions. The stability of the formulations was evaluated by determining particle size, PDI, and zeta potential.

### 2.3. Fourier-Transform Infrared Analysis

Fourier-transform infrared (FT-IR) spectroscopy was performed using a Frontier FT-IR spectrometer (PerkinElmer, Waltham, MA, USA) to establish interactions between the raw material used for NPs formulation (Eudragit^®^ L100 polymer and EOL). Additionally, the nanoformulations (NP-BCO-D, NP-BCO-R, NP-EOL-D and NP-EOL-R) were examined. The polymer and EOL were evaluated directly, while the NPs were centrifuged and the resulting pellet was placed in a desiccator. Spectra were examined over wavenumber range of 4000 to 400 cm^−1^.

### 2.4. Cell Viability Assessment

Cell viability was determined using the Mosmann method with slight modifications [24]. For the assay, Vero cells were cultured in 96-well plates at a density of 1.5 × 10^4^ cells/well in DMEM with 10% FBS and 1% DMSO. After 24 h, the cells were supplemented with different concentrations of EOL, CRV, NP-EOL-R, NP-EOL-D, NP-BCO-R, and NP-BCO-D and incubated for 48 h at 37 °C and 5% CO_2_. Vero cells added to only DMEM with 10% FBS and 1% DMSO were used as negative controls. After 48 h incubation, cell viability was determined by adding 10 μL of 3-(4,5-dimethylthiazol-2-yl)-2,5-diphenyltetrazolium bromide (MTT) solution at a concentration of 5 mg/mL per well, followed by incubation for 3 h at 37 °C in a 5% CO_2_ atmosphere. The culture medium was removed, and 100 μL of DMSO was added. Absorbance was measured at 540 nm using a Thermo Scientific Model 357 microplate reader (Waltham, MA, USA). The cytotoxic concentration 50 (CC_50_) was determined as the concentration of NP-EOL, NP-BCO, CRV, and EOL required to reduce cell viability by 50%, relative to the viability of untreated cells (negative control). Experiments were performed in quadruplicate.

### 2.5. Evaluation of Antiherpetic Activity In Vitro

A viral plaque reduction assay was performed to determine the IC_50_ according to the methodology described by Silva Mares et al. [25]. Briefly, confluent monolayers of Vero cells in 24-well culture plates were incubated with 25 plaque forming units (PFUs) of HSV-1 for 1 h at 37 °C. The supernatant was discarded, and fresh medium supplemented with 0.32% IgG and 1% DMSO was added. Subsequently, concentrations of 11, 22, and 44 μg/mL of NP-EOL, NP-BCO, CRV, and EOL were evaluated; these concentrations were selected based on prior cytotoxicity results, representing low, medium, and high values that were non-toxic. The cells were incubated for 48 h. Finally, the cells were fixed with methanol and stained with Giemsa reagent (CTR Scientific, Monterrey, Nuevo Leon, Mexico). The IC_50_ was determined as the concentration at which a 50% reduction in PFU formation was observed compared with the 0% reduction in the negative control (infected cells). All assays were performed in triplicate. The selectivity index (SI) of each sample was calculated as the ratio of CC_50_ to IC_50_.

### 2.6. Ex Vivo Biophysical Evaluation of Skin Treated with EOL-Loaded NP

The porcine ear skin was obtained from the R.E.T.S.A. slaughterhouse in Monterrey, NL, Mexico. After cleaning and the removal of subcutaneous fat, the skin was frozen at −4 °C for up to four weeks before use.

For the experiment, the skin was thawed and hydrated for 30 min. Subsequently, it was mounted in a modified Franz diffusion cell, consisting of a donor compartment and a receptor compartment, with the porcine skin placed between them. The receptor compartment contained 15 mL of phosphate-buffered saline (PBS) at pH 7.4 and was maintained under magnetic stirring (300 rpm) at 36.0 ± 0.5 °C. The donor compartment (with an area of 2.54 cm^2^) was filled with 1 mL of the treatment of NP-EOL or NP-BCO and covered to prevent evaporation. The formulations were in contact with the skin for 4, 6, and 8 h. After exposure time, the formulations were removed from the skin surface by gently patting with cotton. Subsequently, the effect of the formulations was evaluated in terms of transepidermal water loss (TEWL), skin surface pH, stratum corneum water content (SCWC), as well as the melanin and erythema index, and these were measured on the pig skin with the respective Tewameter TM300, pH 905, Corneometer CM825, and Mexameter MX 18 probes, respectively, all connected to a MPA5 system (Courage & Khazaka, Köln, Germany). Measurements were carried out in quintuplicate, in a draft-free room, with controlled temperature (25 ± 2 °C) and relative humidity (30–40%).

### 2.7. Statistical Analysis

Statistical analysis was performed using GraphPad Prism software version 7.0 (San Diego, CA, USA). One-way ANOVA, followed by a multiple comparison test, was conducted to compare the means of more than two groups (*p* < 0.05).

## 3. Results

### 3.1. Preparation and Characterization of EOL-Loaded Nanoparticles

The NP-EOL-R and NP-EOL-D formulation were obtained using the nanoprecipitation technique established by Fessi et al. [23]. The physicochemical characteristics of the nanoformulations are presented in Table 1.

**Figure 1:**
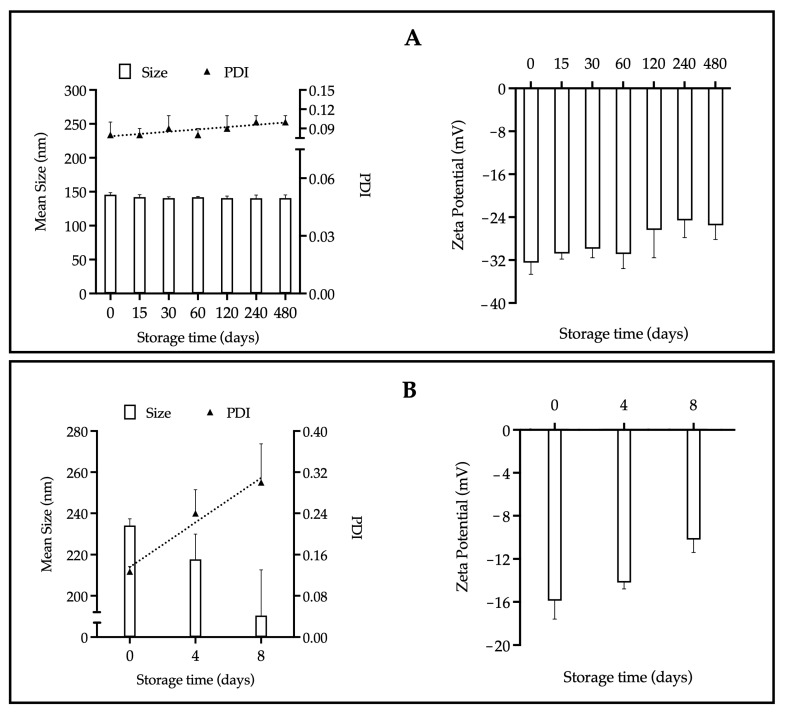
Stability of the NP-EOL-R (**A**) and NP-EOL-D (**B**) as a function of particle size, PDI and zeta potential (Mean ± SD, *n* = 3). The dashed line corresponds to a visual guide to indicate the general direction of the data.

As shown in Figure 1, the stability parameters of the EOL-loaded NPs: particle size, polydispersity index (PDI), and zeta potential, were evaluated over a period of 480 days at 25 °C under relative humidity conditions of 40–60%.

### 3.2. Fourier-Transform Infrared Spectroscopy Analysis of NP-EOL Components

**Figure 2:**
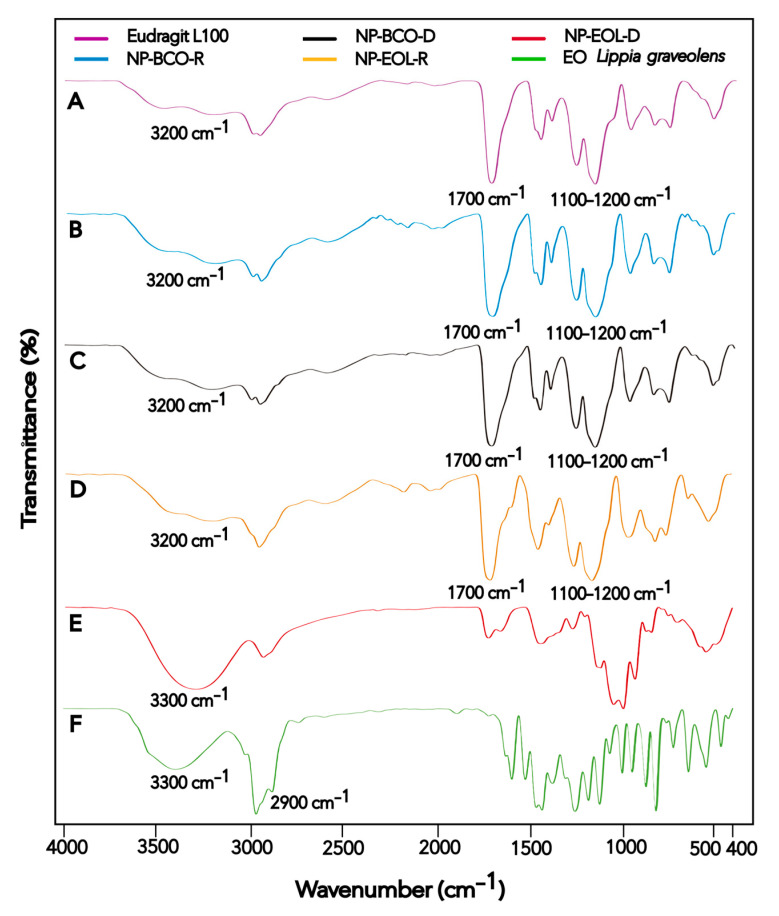
FT-IR spectra of Eudragit^®^ L100 (**A**), NP-BCO-R (**B**), NP-BCO-D (**C**), NP-EOL-R (**D**), NP-EOL-D (**E**), and EO *Lippia graveolens* (**F**).

FT-IR spectroscopy was used to characterize the Eudragit^®^ L100 polymer, *Lippia graveolens* EO, EOL-loaded NPs, and unloaded NPs, in order to analyze the interactions between the NP components. The spectrum of each material is shown in Figure 2.

**Figure 3:**
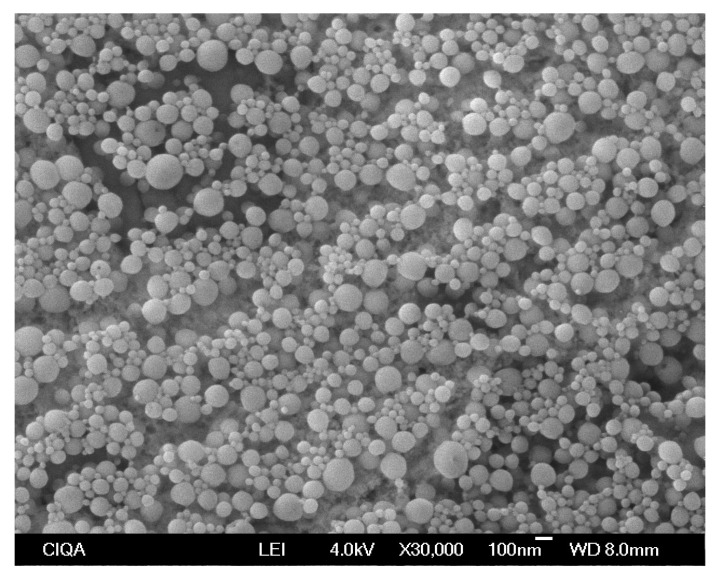
Evaluation of the size and homogeneity of nanoparticles loaded with *Lippia graveolens* essential oil, obtained by the nanoprecipitation technique and purified through evaporation, using scanning electron microscopy (SEM) at a magnification of ×30,000.

The morphology, size, and homogeneity of NP-EOL-R were further analyzed by SEM. Representative micrographs are presented in Figure 3.

### 3.3. Cytotoxicity Against Vero Cells and in Vitro Antiherpetic Activity

The cytotoxic effects of NP-EOL, NP-BCO, EOL, and CRV are shown in Table 2. NP-BCO formulations purified by dialysis and reduced pressure evaporation exhibited no cytotoxicity toward Vero cells at the tested concentrations (>200 μg/mL). The CC_50_ values for NP-EOL-R, NP-EOL-D, EOL, and CRV were 71.89, 57.16, 84.81, and 83.32 μg/mL, respectively.

The antiherpetic activity of NP-EOL, NP-BCO, EOL, and CRV was evaluated using a plaque reduction assay. NP-EOL-R showed the highest antiviral efficacy, with an IC_50_ value of 25.83 μg/mL, compared with NP-EOL-D with an IC_50_ of 36.60 μg/mL. In contrast, EOL and CRV inhibited HSV-1 with IC_50_ values of 47.74 and 27.55 μg/mL, respectively. The SI calculated for NP-EOL-R, NP-EOL-D, EOL, and CRV was 2.78, 1.56, 1.78, and 3.02, respectively.

### 3.4. Ex Vivo Biophysical Evalaution of Skin Treated with EOL-Loaded Nanoparticles

**Figure 4:**
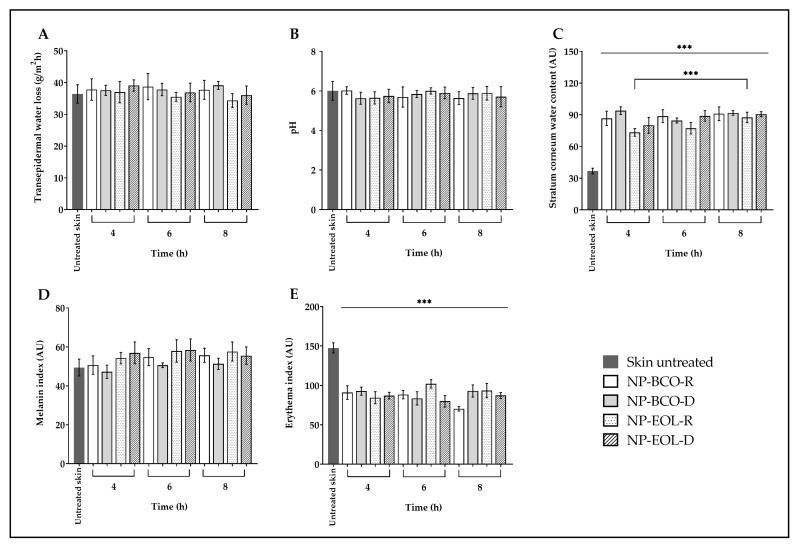
Ex vivo biophysical effect on transepidermal water loss (**A**), pH (**B**), stratum corneum water content (**C**), melanin (**D**), and erythema (**E**) after the contact of EOL-loaded NPs compared with untreated skin (mean ± SD, *n* = 5). *** Indicates significant differences *p* < 0.001, compared to untreated skin.

The biophysical parameters of porcine skin were evaluated before the application of the nanoformulations and after 4, 6 and 8 h of contact time (Figure 4). In the case of untreated skin, TEWL showed no significant changes after exposure to the nanoformulations (baseline value 36.59 ± 2.42 g/m^2^h; Figure 4A). Similarly, the surface pH of the untreated skin (6.09 ± 0.54) remained unchanged following the treatment (Figure 4B). However, a statistically significant increase was observed in the SCWC of treated skin compared with the untreated controls (baseline: 36.04 ± 2.75 AU), after 4, 6 and 8 h of application (Figure 4C). In the case of NP-EOL-R, SCWC values nearly doubled. Regarding the melanin index, the values obtained after 4, 6 and 8 h of exposure to the nanoformulations were like those of the untreated skin (baseline: 50.43 ± 5.59 AU; Figure 4D). Finally, the erythema index decreased significantly after contact with the nanoformulations at all tested time points, compared with untreated skin (baseline: 149.55 ± 6.44 AU; Figure 4E).

## 4. Discussion

In this study, NPs were developed with the aim of improving the topical delivery of oregano EO for the treatment of HSV-1. The NPs were formulated using the nanoprecipitation technique established by Fessi et al., which is widely employed for the encapsulation of hydrophobic compounds such as EOs [26]. This technique is distinguished by its simplicity, rapidity, and versatility, as it is compatible with a broad range of polymers and solvents, offering flexibility in material selection depending on the intended application [27]. Among the commonly used polymeric materials are polymethylmethacrylate derivatives, such as Eudragit^®^ L100. This polymer has been approved by the FDA for the delivery of active compounds targeted to the skin due to its ability to dissolve at pH 6. Frequently, in skin disorders besides inflammation and epidermal lesions, the pH tends to shift to a near neutral environment, this favors the release of the active compound [28,29]. Dong et al. [30], evaluated the skin penetration and release of dexamethasone utilizing a Eudragit^®^ L100-based NP through electron paramagnetic resonance studies. The results showed an in vitro release efficiency and skin penetration of dexamethasone in comparison with commercial cream; the experiments were both conducted on intact and barrier-disrupted skin at pH values above 6.

Once the NPs were obtained, their physicochemical characterization was carried out based on particle size, PDI, and zeta potential (Table 1). The NP-EOL-R formulation exhibited a size of 145.89 ± 5.89 nm. This result is consistent with that reported by Zhang et al. [31], who prepared a Eudragit^®^ L100-based NP loaded with *Piper nigrum* L. EO using nanoprecipitation followed by solvent removal under reduced pressure, obtaining particles with a size of 178 nm. On the other hand, the NP-EOL-D formulations showed a particle size of 232.1 ± 13.50 nm, in an agreement with the findings of Silva-Flores et al. [32], who encapsulated essential oils from *Rosmarinus officinalis* and *Lavandula dentata*, obtaining particle sizes ranging from 227 to 231 nm using the nanoprecipitation technique, followed by purification using dialysis. In our study, the particle size difference, of approximately 80 nm between NP-EOL-R and NP-EOL-D, is attributed to the process used to purify the NPs. Several studies have reported that solvent evaporation rate can influence the physicochemical characteristics of NPs, including their morphology, particle size, and release profile [33,34]. During evaporation, the application of reduced pressure increases the solvent removal rate, generating density differences between the surface and the core of the particles. This phenomenon induces rapid structural relaxation, preventing the full extension of polymer chains and resulting in their contraction, which leads to smaller particles. Conversely, the slower solvent removal rate achieved by dialysis allows sufficient time for polymer chain rearrangement before the particle solidification occurs [35].

The particle sizes obtained for NP-EOL-R and NP-EOL-D fall within the 200 nm range. Donalisio et al. [36], demonstrated that chitosan nanospheres loaded with acyclovir, with a particle size of 200 nm, exhibited 13-fold higher in vitro antiviral activity against HSV-1 compared with non-encapsulated acyclovir. Furthermore, in vitro permeation studies showed that the NPs improved acyclovir deposition in the SC when compared with a commercial cream. In the present study, both NPs formulations exhibited a suitable particle size for deposition in the SC and within skin folds, making them appropriate candidates for the topical delivery of EOs.

The PDI is associated with particle size distribution within a population of NPs. In the Zetasizer Nano-ZS90 (Malvern Instruments), the PDI values range from 0 to 1, where the ones closer to 0 indicate a uniform particle size distribution. In this study, the formulations presented a PDI ≤ 0.2 (Table 1), which falls within the range considered acceptable for polymer-based nanoparticle applications [37]. A homogeneous particle size distribution is a critical attribute that ensures consistent NPs interactions (e.g., bioadhesion and EO release) across the skin surface and with viral particles, potentially enhancing antiviral treatment efficacy.

In this study, the electrokinetic properties of nanoformulations were evaluated. The zeta potential is based on the measurement of the electrochemical equilibrium at the interface (Stern and diffuse layers) surrounding the dispersed NPs [38]. The zeta potential of NP-EOL-R was −32.47 ± 2.18 mV, whereas NP-EOL-D exhibited a value of −15.90 ± 1.70 mV. The negative values indicate that negative charges dominate the diffuse double layer of the NPs, which is attributed to the anionic nature of the Eudragit^®^ L100 polymer due to the ionization of carboxylic end groups exposed on the surface of the polymer chains [28]. The difference in the magnitude of the zeta potential between NP-EOL-R and NP-EOL-D may be due to the higher surface-to-volume ratio of the smaller NP (NP-EOL-R), which favors a greater interaction of the surface charges with ions in the surrounding medium. Moreover, studies on the electrical double layer in low ionic strength media such as distilled water have shown a less compact structure, facilitating more interactions between smaller particles and the medium, thereby increasing the zeta potential [39]. Therefore, this increase in interactions for smaller NPs may explain why the zeta potential value of NP-EOL-R was approximately twice that of NP-EOL-D.

A NPs suspension is considered stable when it maintains its initial physical characteristics (e.g., size, PDI, zeta potential) over a defined period. Visual appearance, such as the absence of aggregates, is another relevant parameter for assessing stability [40]. In colloidal systems, instability may occur when the absolute value of the zeta potential is low, indicating that attractive Van der Waals forces overcome repulsive forces, thus promoting particle aggregation. In contrast, when particles exhibit a high surface charge density or an absolute zeta potential equal to or greater than ± 30 mV, sufficient electrostatic repulsion is generated to prevent coalescence and promote formulation stability [38]. According to the guidelines of the International Council for Harmonisation of Technical Requirements for Pharmaceuticals for Human Use (ICH), the storage stability of nanomedicines must be evaluated for a minimum of 12 months [41,42]. Figure 1 shows the stability of the nanoformulations stored at 25 °C and 40–60% relative humidity; these conditions were chosen based on the recommendations of current antiviral treatments. In this study, NP-EOL-R (Figure 1A) showed no changes in physical characteristics (size, PDI, zeta potential) over 480 days. In contrast, NP-EOL-D (Figure 1B) exhibited an increase in PDI from 0.1 to 0.3 after 8 days of storage, suggesting increased heterogeneity in particle size. Also, the zeta potential of NP-EOL-D decreased to −10 mV, indicating a reduction in electrostatic repulsion forces, which compromised the stability of the formulation and correlated with aggregate formation and phase separation. These results indicate that the storage conditions employed affected the integrity of NP-EOL-D. In contrast, colloidal systems with zeta potentials above ± 30 mV, such as NP-EOL-R, exhibited greater stability. This is consistent with the findings of Zhang et al. [31], who developed a Eudragit^®^ L100-based nanoparticle formulation loaded with *Black pepper* EO, which showed a zeta potential of −57 mV and remained stable for five weeks under various temperature conditions.

As part of the physicochemical characterization of the nanoformulations, the encapsulation efficiency percentage (%EE) was determined using a validated GC-FID method. The %EE values for NP-EOL-R and NP-EOL-D were 16.23 and 29.61%, respectively. These results were lower than those reported by previous reports [22], likely due to differences in the polymer employed or the purification method used.

The FT-IR spectra obtained for the raw materials and nanoformulations are presented in Figure 2. The spectrum of the Eudragit^®^ L100 polymer (Figure 2A) is consistent with the one reported by Saadallah et al. [43]. In the FT-IR spectra, two bands were observed, one near 1156.02 cm^−1^ and another in the region of 1255.91 cm^−1^, both characteristic of C–O stretching vibrations of esters. Additionally, a band appeared around 1711.61 cm^−1^ corresponding to C=O stretching vibrations of carboxylic acids, and another at 3202.42 cm^−1^ associated with hydroxyl group (O–H) vibrations. These signals are characteristic of acrylic copolymers. The signals observed in the Eudragit^®^ L100 polymer spectrum were also present in the spectra of NP-BCO-R and NP-BCO-D (Figure 2B and Figure 2C, respectively), confirming that the structural characteristics of the polymer were preserved in both nanoformulations. On the other hand, the EOL spectrum (Figure 2F) showed a broad band around 3369.87 cm^−1^ and a peak at 2959.55 cm^−1^, which are characteristic of O–H and C–H stretching vibrations, respectively. These signals are consistent with those reported by Gutierrez et al. [44]. The O–H and C–H groups detected in the EOL spectrum may be attributed to the presence of CRV, a component with a phenolic functional group that is found in high proportion in EOL. The spectrum of NP-EOL-R (Figure 2D) was similar to that of NP-BCO-R, supporting the inference that the EOL was successfully encapsulated. Moreover, no new bands corresponding to the formation of new compounds were observed, indicating that only physical or weak chemical interactions occurred between the EOL and the polymeric chains, and that no chemical reactions took place among the NP components. In contrast, the spectrum of NP-EOL-D (Figure 2E) differed from that of NP-BCO-D, this finding suggests that some EOL components may have been retained or adsorbed on the polymer surface [26].

As will be discussed later, NP-EOL-R exhibited favorable biological effects, which could offer a promising alternative for the treatment of HSV-1 infections. In consequence, it was also analyzed by SEM to assess the NPs morphology, size, and homogeneity in the size particles. As illustrated in Figure 3, the NPs exhibited a spherical shape and smooth surface. No particle aggregates were observed, and the particle size (within the 200 nm range) was consistent with the hydrodynamic diameter measured for NP-EOL-R (145.89 ± 5.89 nm).

To evaluate the antiherpetic activity, Vero cells were used as this cell line was approved by the National Committee for Clinical Laboratory Standards for susceptibility tests against various viral infections, including HSV-1 [45]. To explore the potential use of nano-encapsulated *Lippia graveolens* EO as an antiviral agent, the toxicity of the formulation was evaluated in vitro utilizing Vero cells. Cytotoxicity was assessed using the MTT assay for the treatments NP-BCO-R, NP-BCO-D, NP-EOL-R, NP-EOL-D, EOL, and its major component, CRV. As shown in Table 2, at concentrations below 200 µg/mL, NP-BCO purified by reduced pressure evaporation and dialysis did not exhibit cytotoxic effects on Vero cells. This finding aligns with Slavkova et al. [46], who evaluated the cytotoxic effects of a Eudragit^®^ L100 NP on the viability of the human keratinocyte HaCaT cell line, determining that the NPs did not reduce cell viability or exhibit toxic effects. Regarding the EOL, a CC_50_ of 84.81 ± 1.36 µg/mL was obtained. Previously, Gómez et al. [47], reported a comparable cytotoxicity for *Lippia graveolens* EO in the HEK293 cell line, with a CC_50_ of 90.0 ± 18.1 µg/mL. For CRV, a CC_50_ of 83.32 ± 1.17 µg/mL was observed, which is similar to the value reported by Nakamura et al. [48], who obtained a CC_50_ of 86 ± 1.41 µg/mL. Finally, the CC_50_ of NP-EOL-R was 71.89 ± 0.41 µg/mL, while that of NP-EOL-D was 57.16 ± 0.86 µg/mL, the latter showing greater cytotoxicity. In a previous study conducted by the same research group, the compounds present in the EOL were identified [22]. Therefore, the difference in cytotoxicity between the two nanoformulations could be related to the encapsulation of EOL components in different proportions depending on the purification method employed. It is worth noting that the reduced pressure evaporation method favored the encapsulation of compounds such as caryophyllene oxide and butylated hydroxyanisole, unlike dialysis. In a study conducted by Damayantii et al. [49], the in silico toxicity of food preservatives was evaluated, and it was demonstrated that butylated hydroxyanisole exhibited moderate toxicity. Furthermore, another study reported that caryophyllene oxide inhibits the mitochondrial electron transport chain, which could affect cell viability and therefore modify toxicity [50]. However, further studies are needed to establish the cause of the observed toxic effects. Based on the cytotoxicity results in Vero cells, at concentrations of 11, 22, and with 44 µg/mL of NP-BCO-R, NP-BCO-D, NP-EOL-R, and NP-EOL-D, EOL and CRV were selected for subsequent antiviral assays.

The antiviral activity of the nanoformulations against HSV-1 was evaluated using the plaque reduction assay, a widely accepted method to test susceptibility of viruses that can induce cytopathic effects [51]. These effects manifest as structural and functional alterations in infected cells such as cell rounding, enlargement, granulation, syncytia formation, loss of adherence to the substrate, and cell lysis [52,53]. Cellular damage caused by viral replication manifests in the formation of clear zones or plaques in the cell monolayer that become evident after staining the cells. Thus, the plaque reduction assay allows for the determination of the IC_50_ of a substance based on its ability to reduce the number of plaques formed. The IC_50_ values of the tested treatments are presented in Table 2. NP-BCO purified by reduced pressure evaporation and dialysis did not show in vitro antiviral activity against HSV-1 at any of the concentrations tested. In contrast, free CRV exhibited an IC_50_ of 27.55 ± 1.66 µg/mL, a value lower than that reported by Pilau et al. [16], who obtained an IC_50_ of 48.6 µg/mL. The IC_50_ of EOL was 47.74 ± 2.80 µg/mL, which is approximately half the value reported by Pilau et al. (IC_50_ de 99 μg/mL) [16]. Some studies suggest that the antiviral activity of EOs and their components is due to their lipophilic nature, which can induce alterations in viral particles (disruption of the viral envelope or capsid disintegration) and interfere with viral binding to the host-cell receptors [17,54]. Gilling et al. [55], reported that oregano EO at 4% (*v*/*v*) and CRV at 0.5% (*v*/*v*) caused an increase in the particle size of murine norovirus, capsid disintegration, and the subsequent loss of viral infectivity. In another study, pretreatment of HSV-1 virions with CRV at 100 µM resulted in a disruption of 79% of the viral envelope [56]. Another proposed mechanism involves the inhibition of the interaction between the virus and host-cell receptors. In this context, thymol, another major component of EOL, has also been documented for its antiviral properties. In an in silico study, Cura et al. [57] evaluated the interaction of thymol with HSV-1 and showed that hydrogen bonds were formed between thymol and the viral glycoproteins gB (i.e., amino acids GLN416, LEU228, and GLU42) and gD (i.e., amino acids PRO172 and ARG174); these interactions could interfere with viral adhesion and penetration into hosts cells, thereby preventing the early steps of the infection process. On the other hand, the antiherpetic activity of NP-EOL-R showed an IC_50_ of 25.83 ± 1.53 μg/mL, compared with 36.60 ± 2.41 μg/mL for NP-EOL-D, demonstrating greater antiviral effectiveness in the nanoparticles purified by reduced pressure evaporation. This difference could be related to the following factors: (i) The purification method influenced the profile of encapsulated compounds, as reflected in the %EE values obtained (i.e., 16.92% for NP-EOL-R and 29.61% for NP-EOL-D). This variation suggests that each purification method favored the differential encapsulation of active components, thereby impacting the observed antiviral activity. (ii) The surface characteristics of the nanoparticles (zeta potential) may affect their interaction with the virus. In this regard, Yadavalli et al. [58], reported that polyanionic molecules can interact with the cationic domains of viral glycoproteins (gB, gC). As noted previously, NP-EOL-R exhibited an absolute zeta potential nearly twofold greater than that of NP-EOL-D. This higher negative surface charge may contribute to enhanced electrostatic interactions with the virus, thereby facilitating the release of EOL and its interaction with the viral envelope. However, no experimental evidence is currently available to confirm this mechanism. Therefore, additional studies including release profiling, cellular uptake assays, and antiviral activity evaluation at different stages of the viral replication cycle are required to elucidate the mechanism responsible for the greater activity observed.

The SI is a parameter that indicates a compound’s ability to inhibit a virus without significantly affecting host cells. It is calculated as the ratio between the cytotoxic concentration (CC_50_) and the effective antiviral concentration (IC_50_). A SI value equal to or greater than 4 suggests effective antiviral activity with low cytotoxicity, making the compound suitable for therapeutic use [59]. The SI values obtained in this study are presented in Table 2. As shown, the EOL exhibited a SI value of 1.78, while NP-EOL-R and NP-EOL-D had values of 2.78 and 1.56, respectively. These results indicate that encapsulating EOL in nanoparticles, combined with solvent removal by reduced pressure evaporation, increased the SI value, thereby improving both the safety and efficacy of EOL as an antiviral agent. Although the SI value obtained for NP-EOL-R did not reach the reference threshold (SI ≥ 4), it is important to emphasize that this formulation improved selectivity compared with the free EO. Nevertheless, the SI value of below 4 highlights the need to optimize the nanoformulation. In this regard, adjustments in the preparation conditions (e.g., solvents and polymer in the organic phase) and in the purification method could modify the profile of encapsulated and non-encapsulated compounds. Such modifications may reduce the contribution of components responsible for immediate cytotoxicity, thereby improving the balance between antiviral efficacy and safety.

The in vitro assays conducted in this study allowed for a controlled, rapid, and cost-effective evaluation of the antiviral potential of EOL encapsulated in NPs, as well as their effects on cell viability. However, the validation of these results in HSV-1 in vivo infection models is required, since such models offer a more complete perspective on efficacy, dosage, pharmacokinetics, and safety within a more complex biological system.

On the other hand, evaluating the effect of topical treatments using a porcine skin model is a versatile, efficient, and predictive approach that supports the development of new formulations. Because porcine skin is akin to human skin in various morphophysiological characteristics, such as vascular organization, lipid film composition, epidermal and SC thickness, and follicular structure, it is a suitable option for studying skin damage, wound healing, and drug delivery [60]. Another advantage is that porcine skin can be obtained as a slaughterhouse by-product, which helps address ethical concerns related to animal experimentation while also reducing experimental costs [61].

Biophysical parameters such as TEWL, pH, water content, and the erythema and melanin index are among the most used indicators to assess the skin barrier function.

TEWL measurement is widely used in dermatological research as an indicator of skin barrier integrity as it calculates the amount of water that diffuses from the dermis and epidermis through SC to the skin surface [62]. When the skin is damaged, as in the case of skin disorders or burns, TEWL levels may increase, indicating impaired barrier function [63].

As shown in Figure 4A, TEWL values did not differ significantly from untreated skin following the application of NP-EOL-R, NP-EOL-D, NP-BCO-R, and NP-BCO-D on the skin surface for 4, 6, and 8 h. These findings suggest that the water-based composition of the nanoformulations did not compromise the skin’s hydrolipidic barrier. Similar results were reported by Saraiva et al. [64], who demonstrated that in vivo application of chitosan nanoparticles loaded with *Helichrysum italicum* EO on human skin did not significantly alter TEWL.

Another important parameter in maintaining skin barrier function is pH. In humans, the skin has an acidic pH ranging from 4.1 to 5.8, depending on the body site. Skin pH influences various functions, including keratinocyte differentiation and the formation and function of the lipid bilayer [65]. As shown in Figure 4B, skin pH values did not change significantly after contact with the nanoformulations for 4, 6, and 8 h. These findings are consistent with ex vivo studies by Silva-Flores et al. [66], who reported that the application of Eudragit^®^ EPO nanoparticles loaded with *Rosmarinus officinalis* and *Lavandula dentata* EO caused no significant changes in skin pH on porcine skin. Previous studies have shown that formulations with an acidic pH help maintain the skin’s natural acid mantle. In the present study, the applied nanoformulations did not alter skin pH, suggesting that they preserve the acidic environment essential for microbiome balance, thereby creating unfavorable conditions for the growth of pathogenic bacteria and fungi.

It is well known that the water content of the SC (SCWC) plays a crucial role in the appearance and health of the skin, as it influences its elasticity, flexibility, and functional properties such as active ingredient permeation. The SC normally contains between 10 and 30% water [67]. However, various factors, including environmental exposure, surfactant use, solar radiation, or skin disorders, can reduce the SCWC to below 10%, affecting the enzymatic activity required for normal desquamation. This may lead to decreased flexibility and a dry, scaly appearance of the skin [68]. Figure 4C shows the SCWC values following contact of the nanoformulations with the surface of porcine skin. A statistically significant increase in SCWC levels was observed compared with untreated skin. This increase may be attributed to the natural moisturizing factor, which is composed of amino acids and their derivatives, such as pyrrolidone carboxylic acid and urocanic acid, as well as lactates, urea, and electrolytes. These components possess hydroscopic properties that may help retain the water present in the nanoformulations, thereby contributing to skin hydration [69]. Furthermore, given that the nanoformulations are an aqueous suspension containing more than 99% (w/w) water, this characteristic may, at least in part, account for the increase in the SCWC observed after their application. Notably, the SCWC values increased progressively with longer contact times of NP-EOL-R with the skin, suggesting that the nanoformulation may promote sustained SC hydration, which is promising for the development of topical systems with prolonged activity.

On the other hand, skin color changes can be evaluated as indicators of skin barrier integrity. Human skin color is primarily determined by the presence of melanin, hemoglobin, carotenoids, and bilirubin [70]. Melanin not only contributes to pigmentation but also protects the skin against oxidative stress induced by UV radiation, air pollutants, and chemical compounds [71,72]. Melanin content varies with sex, age, ethnicity, lifestyle, environmental conditions, and exposure to certain substances [73,74]. In an in vivo study, the melanin index of the skin was evaluated using a Mexameter probe after applying a cream containing *Olea europaea* leaf extract. These results indicated no significant changes in pigmentation after two months of treatment [75]. These findings are consistent with the results shown in Figure 4D, where no statistically significant differences in the melanin index were observed between the skin treated with the nanoformulations and untreated skin, suggesting that the formulations do not alter melanin content on the skin surface. Moreover, the melanin index remained unchanged for up to 8 h post-application, indicating that the nanoformulations do not induce changes in skin melanin levels even after prolonged contact.

Regarding hemoglobin, which is present in red blood cells circulating through blood vessels, it is associated with the red coloration of the skin because is strongly reflects longer wavelengths while predominantly absorbing radiation in the shorter wavelength range (corresponding to blue) [76]. Under certain conditions, such as contact with allergens, viral infections, or increased body temperature, vasodilation is triggered, increasing blood flow to the superficial capillaries of the skin. The accumulation of hemoglobin in these areas manifests as skin redness, known as erythema [77]. Alam et al. [78], used the same Mexameter probe in an in vivo model to evaluate the erythema index after applying a cream containing *Echinacea purpurea* extract. Their results showed no increase in erythema levels during the study period. As shown in Figure 4E, a statistically significant decrease in erythema index values was observed following the application of the nanoformulations compared with untreated skin. This result may suggest that the deposition of nanoparticles on the skin surface, due to their nanometric size and solid nature, favors the dispersion of incident light. Puentes Ossa et al. [79], reported that conjugated fluorene silica nanoparticles sized between 100 and 125 nm are capable of scattering light and exhibit low absorption coefficients within the spectral range 300–800 nm, which encompasses the wavelengths used by the Mexameter (568–880 nm). Therefore, the amount of radiation absorbed by hemoglobin would be modified by the presence of the NPs, resulting in an apparent decrease in erythema values.

To our knowledge, the antiviral activity of EOL in NPs has not been previously reported.

## 5. Conclusions

In the present study, NP-EOL were obtained with adequate physicochemical characteristics for topical use. The results suggest that nanoencapsulation combined with purified evaporation under reduced pressure potentiated the antiherpetic activity of *Lippia graveolens* EO by about two times, and an improvement in the security profile. The biophysical evaluation in skin demonstrated that the nanoformulations favors SC hydration without compromising the skin barrier. Therefore, NP-EOL is a promising alternative for topic treatment against viral infections caused by HSV-1.

## Figures and Tables

**Table 1:** Physicochemical characterization of EOL-loaded NPs (Mean ± SD, *n* = 3).

Formulation	Mean Size (nm)	PDI	Zeta Potential (mV)	pH	%EE
NP-EOL-R	145.89 ± 5.89	0.075 ± 0.024	−32.47 ± 2.18	3.30 ± 0.04	16.92 ± 1.49
NP-EOL-D	232.10 ± 13.50	0.127 ± 0.010	−15.90 ± 1.70	3.18 ± 0.12	29.61 ± 2.26

NP-EOL-R, EOL-loaded NPs purified by evaporation; NP-EOL-D, EOL-loaded NPs purified by dialysis; %EE, encapsulation efficiency percentage, was determined by a previously validated GC-FID method.

**Table 2:** Cytotoxic effect against Vero cell lines, using MTT assay, and antiherpetic activity of polymeric nanoparticles loaded with EOL.

	Vero Cells CC_50_ (μg/mL)	HSV-1 IC_50_ (μg/mL)	SI
NP-EOL-R	71.89 ± 0.41	25.83 ± 1.53	2.78
NP-EOL-D	57.16 ± 0.86	36.60 ± 2.41	1.56
NP-BCO-R	>200	ND	ND
NP-BCO-D	>200	ND	ND
EOL	84.81 ± 1.36	47.74 ± 2.80	1.78
CRV	83.32 ± 1.17	27.55 ± 1.66	3.02

NP-EOL-R, EOL-loaded NPs purified by evaporation; NP-EOL-D, EOL-loaded NPs purified by dialysis; NP-BCO-R, NPs without EO purified by evaporation; NP-BCO-D, NPs without EO purified by dialysis; EOL, essential oil *Lippia graveolens*; CRV, carvacrol majority compound of EOL; SI, selectivity index (CC_50_/IC_50_); CC_50_, concentration which has a 50% inhibitory effect on cells (Mean ± SD, *n* = 4); IC_50_, concentration which has a 50% reduction in PFU (Mean ± SD, *n* = 3); ND, not determined.

## Data Availability

The original contributions presented in the study are included in the article, further inquiries can be directed to the corresponding author.

## Abbreviations

The following abbreviations are used in this manuscript:

HSV-1	herpes simplex virus type 1
NPs	polymeric nanoparticles
SC	stratum corneum
EO	essential oil
CRV	carvacrol
NP-EOL-R	*Lippia graveolens* essential oil-loaded nanoparticles purified by evaporation
NP-EOL-D	*Lippia graveolens* essential oil-loaded nanoparticles purified by dialysis
NP-BCO-R	nanoparticles without essential oil purified by evaporation
NP-BCO-D	nanoparticles without essential oil purified by dialysis
PDI	polydispersity index
FT-IR	Fourier-transform infrared spectroscopy
SEM	scanning electron microscopy
IC_50_	inhibitory concentration 50
CC_50_	cytotoxic concentration 50
SI	selectivity index
TEWL	transepidermal water loss
SCWC	stratum corneum water content
